# Investigation of Performance of Anion Exchange Membrane (AEM) Electrolysis with Different Operating Conditions

**DOI:** 10.3390/polym15051301

**Published:** 2023-03-04

**Authors:** Adam Mohd Izhan Noor Azam, Thuushren Ragunathan, Nurul Noramelya Zulkefli, Mohd Shahbudin Masdar, Edy Herianto Majlan, Rozan Mohamad Yunus, Noor Shahirah Shamsul, Teuku Husaini, Siti Nur Amira Shaffee

**Affiliations:** 1 Fuel Cell Institute, Universiti Kebangsaan Malaysia, Bangi 43600, Selangor, Malaysia; 2 Department of Chemical & Process Engineering, Faculty of Engineering & Built Environment, Universiti Kebangsaan Malaysia, Bangi 43600, Selangor, Malaysia; 3 Petronas Research Sdn Bhd (PRSB), Jalan Ayer Hitam, Kawasan Institusi Bangi, Bandar Baru Bangi 43000, Selangor, Malaysia

**Keywords:** anion exchange membrane (AEM) electrolysis, parametric study, electrolysis performance, hydrogen production, energy efficiency

## Abstract

In this work, the performance of anion exchange membrane (AEM) electrolysis is evaluated. A parametric study is conducted, focusing on the effects of various operating parameters on the AEM efficiency. The following parameters—potassium hydroxide (KOH electrolyte concentration (0.5–2.0 M), electrolyte flow rate (1–9 mL/min), and operating temperature (30–60 °C)—were varied to understand their relationship to AEM performance. The performance of the electrolysis unit is measured by its hydrogen production and energy efficiency using the AEM electrolysis unit. Based on the findings, the operating parameters greatly influence the performance of AEM electrolysis. The highest hydrogen production was achieved with the operational parameters of 2.0 M electrolyte concentration, 60 °C operating temperature, and 9 mL/min electrolyte flow at 2.38 V applied voltage. Hydrogen production of 61.13 mL/min was achieved with an energy consumption of 48.25 kW·h/kg and an energy efficiency of 69.64%.

## 1. Introduction

The recent need for increased environmental protection has led to increased research into eco-friendly green technologies. Green technologies are important in reducing a country’s pollution and can contribute to the economic growth of the nation. One such technology is the use of hydrogen [1]. Hydrogen gas (H_2_) is a clean source of renewable energy, which is mostly separated from other elements such as water (H_2_O) [2]. Electrolysis of water has become one of the sources used for obtaining ultra-pure hydrogen (up to >99.999%) in a non-polluting manner. Electrolysis is used to separate hydrogen molecules from water by passing through electrical energy, thus splitting water into its elements [3,4].

In recent years, studies have shown great progress in improving the performance of electrolysis. They aimed at reducing the consumption of electricity and increasing the production of hydrogen at the same time. Electrolysis efficiency is the efficiency with which the electrolysis converts electricity into hydrogen. It is equal to the energy content (based on the higher heating value) of the hydrogen produced, divided by the amount of electricity consumed. For example, the higher heating value of hydrogen is 142 MJ/kg, which is equal to 39.4 kW·h/kg. So, electrolysis that consumes 50 kW·h of electricity to produce one kilogram of hydrogen has an efficiency of 39.4 kW·h/kg, divided by 50 kW·h/kg, which equates to 79% [5].

There are four main types of electrolysis: alkaline electrolysis, proton exchange membrane (PEM) electrolysis, anion exchange membrane (AEM) electrolysis, and solid oxide electrolysis (SOEL). The alkaline electrolysis and the proton exchange membrane (PEM) electrolysis are well-proven technologies and have been used for commercial electrolytic hydrogen production. The solid oxide electrolysis (SOEL) and the anion exchange membrane (AEM) electrolysis are still in the developmental stage and questions as to their viability remain unanswered [6,7]. The hydrogen from electrolysis can be produced at any location and time when needed, which would be very useful if integrated into a well-built system [8].

The PEM electrolyzer uses IrO_2_ and Pt black as anode and cathode catalysts with an acidic polymer membrane to prevent gas crossover and enable high-pressure working conditions. However, Nafion-based membranes are expensive, and cation contaminants can degrade the system over time [9]. AEM electrolysis cells use anion exchange membranes and two transition metal catalyst-based electrodes with distilled water or low-concentration alkaline solutions. AEM electrolysis technology is still in development and needs more research to enhance power efficiency [10], membrane stability, ionic conductivity [11], stack cost, and catalyst integration [12]. PEM and AEM electrolysis are expected to account for a larger portion of the global hydrogen market due to their environmental advantages. Further research is needed to increase the longevity and durability of AEMs and enhance their resistance to reactive oxygen species. Laboratory-developed AEMs have demonstrated comparable performance levels to commercial AEMs, indicating significant opportunities for progress.

Several challenges have been encountered that have impeded the commercialization of electrolysis. Among the common problems are mass transport, incomplete electrochemical reactions, and the cost of producing the electrolysis [13,14]. In terms of mass transport, a crossover of hydrogen and oxygen ions in the water of the electrolysis stack is one of the most commonly observed problems, which lowers the performance of electrolysis and also results in lower hydrogen energy yields [13]. The high material cost used in components such as catalysts in the membrane electrode assembly (MEA) of electrolysis such as PEM and AEM is a barrier to commercialization. Although the components of most electrolysis probably can be changed or altered, the operating parameters absolutely can be changed in order to optimize the operating conditions for achieving better performance. Such parameters include the electrolyte flow rate, the operating temperature of the electrolysis stack, and the concentration of electrolyte [15,16].

In an AEM electrolysis, changing the input solution to an alkaline electrolyte like KOH or K_2_CO_3_ can significantly increase ion transfer, reducing ohmic resistance and improving reaction kinetics in the MEA. However, higher pH electrolytes typically result in better AEMWE activity but come with increased capital costs due to enhanced causticity [17]. To achieve a balance between activity and cost, a mild alkaline solution is ideal. Nakano et al. found that a 10 wt% K_2_CO_3_ solution outperformed a 10 mM KOH solution due to its lower cell resistance [18], while Kiessling reported that carbonates solution had a self-purging effect and outperformed hydroxides solution at the same pH and high current density [19]. However, the long-term stability of AEMWE in this mild alkaline condition remains unknown.

When the concentration of the electrolyte was increased from 0.5 to 1 M KOH, it reduced resistance and thus reduced the voltage supplied to the stack. At lower cell voltage, the charge transfer resistances are higher than the ohmic resistances. Hence, it appears that at lower KOH concentrations the charge transfer resistance dominates, while at higher concentrations the ohmic resistance dominates. At higher concentrations, it has been shown that the cell stack efficiency increases, enabling production of a large volume of hydrogen by low amount of energy. This reduces the overall cost of the electrolysis performance and makes it more economical [20]. Moreover, on the anode side of AEM electrolysis, a KOH electrolyte solution containing water molecules transporting OH^−^ ions is cycled. Flow of liquid electrolytes is crucial because it is linked to the mass transfer of the redox process. It is essential to comprehend the relationship between flow rate and resistances. The electrolyte inlet flow rate also slightly impacts the efficiency of H_2_ generation via electrolysis [21].

AEMWE performance is enhanced at elevated temperatures due to accelerated electrode reaction kinetics, as well as promoted electron, ion, and mass transportation [22]. Additionally, the thermodynamic potential of the water-splitting reaction decreases by about 8.5 mV per 10 °C [23]. These factors make elevated temperature AEMWE desirable, as demonstrated by Park et al. who optimized the operating temperatures of AEM cells from 50 to 70 °C in 1 M KOH and found that higher temperatures improved performance [24]. At 70 °C, an ultrahigh current density of 1.5 A cm^−2^ was achieved at only 1.9 V, with decreased ohmic resistance confirmed by EIS. This is a result of the increased reaction rate of the electrode and the improved ionic conductivity of the electrolysis. When the kinetic energy of water molecules increases, the amount of energy required to split water molecules into hydrogen decreases [25]. This condition minimizes the cost of hydrogen production from an economic standpoint. However, most current AEMs have poor thermal stability and cannot operate above 70 °C for long periods, limiting their development. Recently, Yan and colleagues developed a poly(aryl piperidinium)-based AEM with low swelling ratio that enabled continuous and stable operation at 80 °C for over 160 h, achieving a high performance of ~1 A cm^−2^ at 1.8 V for pure water electrolysis with a self-supported F-NiFeOOH anode [24].

Moreover, AEM electrolysis can directly produce high purity and pressurized hydrogen, potentially reducing follow-up costs. Ito et al. confirmed successful operation of AEMWE at pressures < 10 bars, finding that increasing H_2_ pressure to 8.5 bars at only the cathode side during operation did not affect electrolysis performance, but helped reduce the humidity of produced H_2_ [26]. They also performed theoretical analyses to confirm the feasibility of operating the pressurized PEM/AEM water electrolysis technology at around 10 bars [27]. However, limitations and risks arise as pressure increases beyond 10 bars [28]. H_2_ cross-permeation through the membrane becomes a serious problem, causing unavoidable hydrogen oxidation reaction at the anode. Each component of the hardware, including the cell, tubing, gas collection, and connections, must meet higher requirements for harsh operating conditions. Therefore, more efforts and breakthroughs are required to narrow the gap between laboratory- and industrial-scale production of cost-efficient hydrogen.

Therefore, the purpose of this work is to determine the effect of operating parameters, such as the inlet concentration of electrolyte, operating temperature, and electrolyte flow rate, on the performance of an anion exchange membrane (AEM) electrolysis. Electrolysis performance was evaluated by measuring the hydrogen production and by analyzing the energy consumption as well as its efficiency, with different current densities over the operating period. The relationship between the operating parameters and the energy efficiency of the AEM electrolysis would be discussed based on hydrogen production.

## 2. Methodology

### 2.1. Experimental Setup

**Figure 1:**
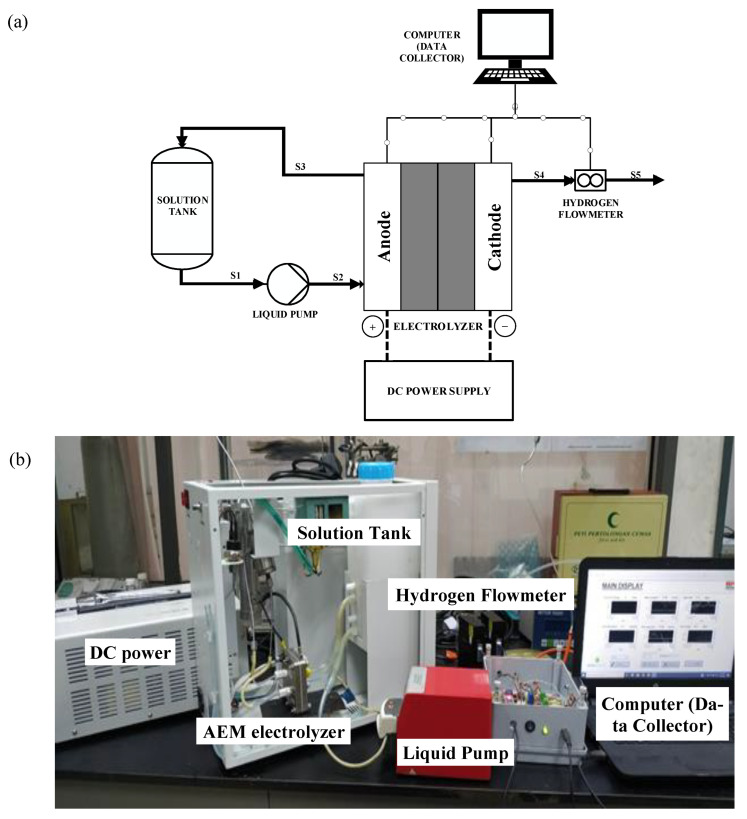
AEM electrolysis setup. (**a**) Schematic diagram. (**b**) Actual photo.

A single cell of AEM type electrolysis cell was used with 5 cm^2^ active area of electrode. For the electrode, catalyst loading with 2 mg/cm^2^ Ni-based catalyst on nickel mesh was used for both anode and cathode. Meanwhile, Sustainion^®^-type AEM was used as a solid electrolyte membrane in the AEM electrolysis. Moreover, a grooved 5 cm² nickel plate with serpentine type flow-field was used at the anode and cathode side. O-ring seals and Teflon gaskets were utilized as a seal, to prevent gas and liquid leakage. A 1 L HDPE-type solution tank (with different KOH concentration) with circulating water was used. A double-headed peristaltic pump (model: BT 120S pump, Falmouth, UK) circulated the electrolyte from the reservoir. Preheated 1 M KOH solution was continuously circulated using the pump through the anode side at a flow rate of 6 mL/min. The electricity for the electrolysis experiments was supplied through a DC power supply. (VOLTEQ HY30100EX). The temperature was maintained at 60 °C throughout, with a water heating circulating system. Figure 1a shows the AEM electrolysis setup schematic, while Figure 1b shows an actual photo of experimental setup.

### 2.2. Single Cell Operation

A commercial AEM electrolysis cell with a 5 cm^2^ active area was used in the experimental setup as shown in Figure 1a,b for performance and operating tests. A 0.5–2.0 M KOH solution was used as an electrolyte. The electrolyte was circulated from a reservoir through the anode side by a peristaltic pump with the flow rate control from 1–9 mL/min. The current supply was controlled from 2.5–6 A which about 0.5–1.2 A/cm^2^ of current density. The temperature of the operational AEM stack was within the range of 30–60 °C. The electrolysis system was operating at room temperature and at atmospheric pressure. The voltage, current, hydrogen flow rate, and temperature were recorded automatically by the computer. The hydrogen produced was collected and analyzed using a mass flowmeter.

### 2.3. Measurement of Cell Efficiency

The reaction at the anode and cathode for the electrolysis in the AEM electrolysis [29] is shown below:

(1) $$\mathrm{Anode}:\;\;2\mathrm{OH}\to \frac{1}{2}O_{2}+H_{2}O+2e^{-}$$

(2) $$\mathrm{Cathode}:\;\;2H_{2}O\to 2H^{+}+2{\mathrm{OH}}^{-}$$

The cell efficiency was measured using efficiency coefficient in percentage based on electrolysis thermodynamic (voltaic efficiency), as shown in Equation (3), and based on specific energy density or energy consumption, i.e., kW·h/kg H_2_, as shown in Equation (4). In Equation (3), the value of 1.48 V is the thermoneutral voltage at which water dissociation takes place, and hence, hydrogen and oxygen are produced with 100% thermal efficiency. This high heating value (HHV) was approached for the thermodynamic electrolysis by considering the water in liquid formation conditions due to the low operating temperature. The cell efficiency coefficient in percentage can be calculated based on the ratio of theoretical thermoneutral voltage, 1.48 V versus actual electrolysis cell voltage during cell operation.

(3) $$Efficiency\;\left(\%\right)=\frac{\;1.48}{Actual\;cell\;voltage}\times 100$$

Energy consumption for the AEM electrolysis unit is calculated based on the amount of used energy in kW·h per amount of hydrogen produced in 2 h, as shown in Equation (4):

(4) $$Energy\;consumption\;for\;electrolysis\;unit\;\left(kW\cdot h⁄kg\right)=\frac{\left(Amount\;of\;Energy\;used\;for\;electrolysis\;unit\left(kW\cdot h\right)\right)}{(Amount\;of\;hydrogen\;produced\;in\;1\;hour\;\left(Kg\right)}$$

The energy efficiency for the AEM electrolysis unit to produce 1 kg of hydrogen can be determined by Equation (5):

(5) $$\mathrm{Energy}\;\mathrm{efficiency}\;(\%)=\frac{Theoretical\;energy\;consumption/1\;kg\;H_{2}@\;STP\;\left(39.4\;kW\cdot h/kg\;based\;on\;HHV\right)}{Calculated\;energy\;consumption\;for\;electrolysis\;unit/1\;kg\;of\;H_{2}}$$

## 3. Results and Discussion

### 3.1. Effect of Electrolyte Concentration of KOH

**Figure 2:**
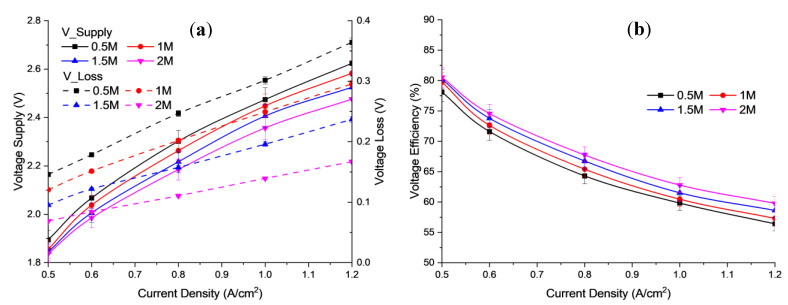
Profile of (**a**) Polarization curve; (**b**) Voltage efficiency, for different alkaline concentrations at 6 mL/min flow rate and 30 °C operating temperature.

Figure 2a shows the polarization curve obtained from the results of the study for the influence of the concentration of potassium hydroxide (KOH) electrolyte in the range 0.5, 1.0, 1.5, and 2.0 M on cell voltage at an operating temperature of 30 °C and 1 bar. Meanwhile, Figure 2b shows the voltage efficiency obtained from the results of the study.

Based on the Figure 2, the concentration of KOH electrolytes on the anode and cathode significantly affects the overall electrolysis efficiency. The results of this experiment are quite close to those reported by Liu et al., who obtained a voltage of 1.9 V under similar conditions. However, in this experiment, the voltage obtained was 2.1 V at a current density of 0.5 A/cm^2^, using 1 M KOH [30]. The performance curves revealed that the cell voltage loss decreased with an increase in the concentration of the liquid electrolyte. There was a considerable increase in AEM electrolysis performance when the concentration was increased from 0.5 to 2.0 M KOH. The cell voltage of electrolysis obtained at a current density of 1.2 A/cm^2^ was 2.48 V for 2.0 M KOH, while the cell voltage was 2.62 V for 0.5 M KOH. These results indicate that the higher concentration of alkaline solution results in a very good ionic conductivity of the membrane (in the MEA), and thus reduces the voltage supplied to the electrolysis process [31].

Moreover, the lower concentration of hydroxide ions can minimize the formation of unwanted by-products, such as chlorates and perchlorates, which can arise at high alkaline concentrations in traditional electrolysis processes. Hence, it would affect the active site of the electrode, and reduce the cell performance.

**Figure 3:**
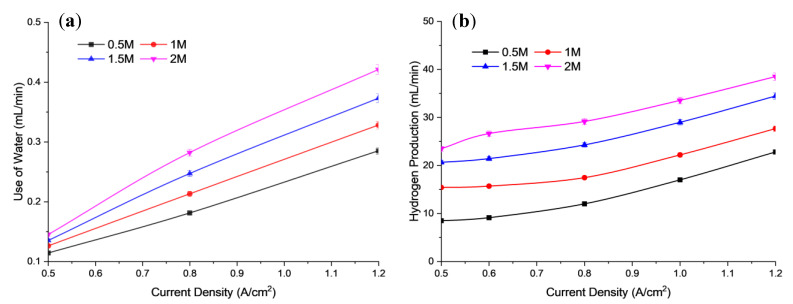
Profile of (**a**) use of water, (**b**) Hydrogen production for different alkaline concentrations at 6 mL/min flow rate and 30 °C operating temperature.

The electrical conductivity of the electrolyte solution played an important role in the electrolysis system. This is evidenced by the increase in electrolyte concentration and current density contributing to the increase in the power supply vector. The increase in required input voltage also led to increased electrical conductivity. This is because the rate of effective ionization has occurred. As a result, H_2_ gas production increased. Figure 3a,b show the use of water and hydrogen production, based on the AEM electrolysis.

In Figure 3a, the measured water amount was collected as an indicator for the use of water during cell operation at different conditions. In this study, the amount of used water can be divided into several parts, including water consumption for electrochemical reaction, water electro-osmotic drag, water diffusion to the cathode, and water losses during water evaporations. Hence, based on calculation, the measured water used in this study did not represent the hydrogen generation in the cell. This means that the obtained water used did not entirely convert hydrogen and oxygen.

**Figure 4:**
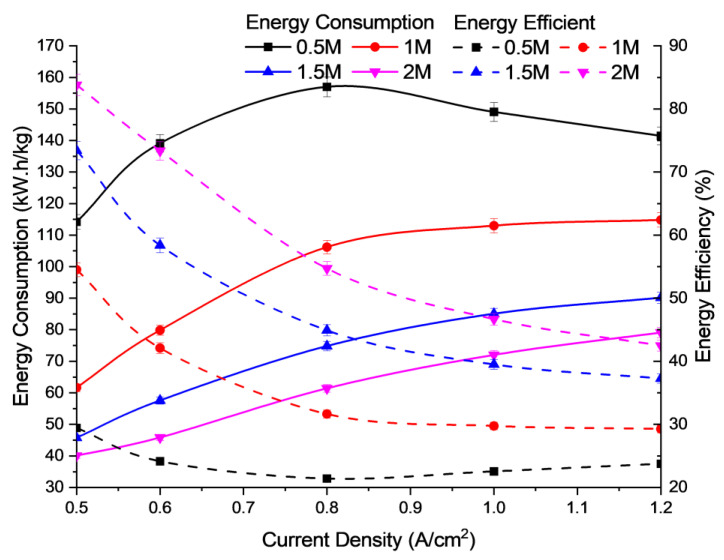
Profile of energy consumption and energy efficiency of electrolysis performance for different alkaline concentrations at 6 mL/min flow rate and 30 °C in operating temperature.

In Figure 3, the data show an increase in H_2_ production rates. Electrolysis depends not only on the number of ions present in the electrolyte solution but also on the mobility of the ions in the solution. In addition, increasing the concentration of electrolyte (KOH) will gradually increase the number of ions in the solution, which subsequently decreases the mobility of the ions due to obstruction [32]. Based on Figure 3, it is observed that increasing the solution concentration will increase the use of water and the hydrogen production rate for better electrical performance due to the relatively high electrolyte concentration range and low supplied voltage. This could be due to the higher concentration of electrolytes releasing more ions into the aqueous medium [33]. Figure 4 shows the energy consumption and energy efficiency against the operating current density at different concentrations of KOH electrolyte solution. There are irregular interactions between cations and anions that limit the free movement of ions across their electrodes, which could influence the energy efficiency. When the concentration of the KOH electrolyte solution is increased, the energy consumed decreases due to the increased energy efficiency used by the electrolysis unit. This result is supported by a study conducted in previous research by Buelvas et al. [34]. Based on Figure 4, it was obtained that the cell performance improves with a slight increase in KOH concentration due to the drastic reduction in ohmic resistance of the AEM and fast reaction kinetics, in which 2 M KOH showed the best performance. The lower concentration of hydroxide ions, i.e., below 2 M, can minimize the formation of unwanted by-products, such as chlorates and perchlorates, which can arise at high alkaline concentrations in conventional alkaline electrolysis processes. Hence, it would affect the active site of the electrode, and reduce the cell performance.

### 3.2. Effect of Operating Temperature

**Figure 5:**
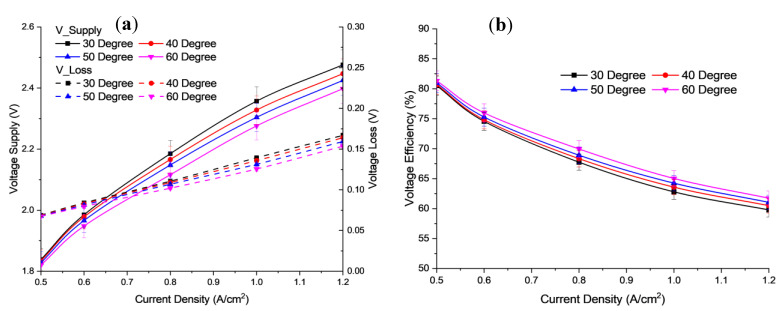
Profile of (**a**) Polarization curve, (**b**) Voltage efficiency for different operating temperatures at 6 mL/min flow rate and concentration of electrolyte 2.0 M KOH.

Figure 5a shows a comparison of polarization curves at 30–60 °C obtained in this work. Figure 5b shows a comparison of voltage efficiency at 30–60 °C. Initially, cell voltage shows an increase as the temperature decreases. Then, for 30 °C and 40 °C, the cell voltage increases slowly when the current density exceeds 0.6 A/cm^2^. At 60 °C, the voltage efficiency is higher than the cell voltage efficiency at 50, 40, and 30 °C. A high temperature can reduce the energy required to break water molecules and increase the electrochemical reaction activities. Moreover, increasing the temperature can also reduce the electrical resistance to different currents [35]. Hence, a demand for the required voltage decreases as the temperature increases at a certain concentration. This results in better performance of electrolysis cells due to the low energy requirements of H_2_ production. As the temperature increases, the ability of the molecule to rupture decreases. The ionic conductivity of the electrolyte and the reaction surface also increase [36]. Based on the experiment, the value of the parameter has been applied to this model. The comparison of polarization curves for the studies is shown in Figure 5a,b based on different temperatures.

**Figure 6:**
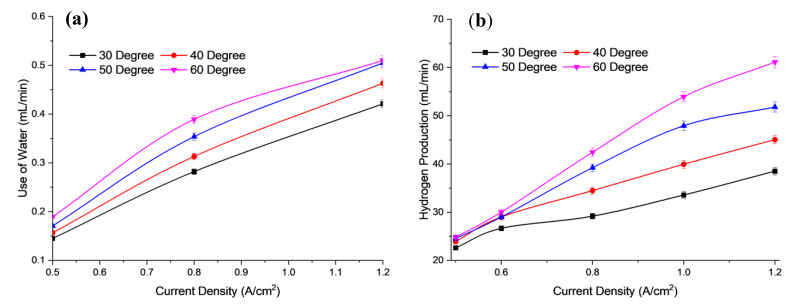
Profile of (**a**) use of water, (**b**) Hydrogen production based on electrolysis efficiency for different operating temperatures at 6 mL/min flow rate and concentration of electrolyte 2.0 M KOH.

Figure 6 shows the use of water and the hydrogen production at different operating temperature of electrolysis. Based on Figure 6a,b, the use of water is directly proportional to the hydrogen production for different operational temperatures of the electrolysis stack. When the use of water at 60 °C is 0.51 mL/min, the hydrogen production is also highest at 61.134 mL/min. The main reason for the increased production is due to increased mobility of the ions within the membrane, which is faster at higher temperatures.

**Figure 7:**
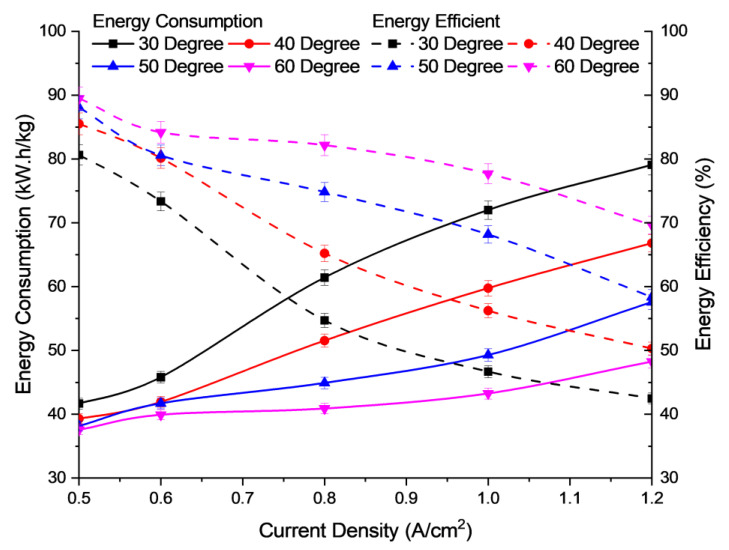
Profile of energy consumption and energy efficiency of electrolysis performance, for different operating temperatures at 6 mL/min flow rate and concentration of electrolyte 2.0 M KOH.

At higher temperatures, the mobility of OH^−^ ions is high due to their loosely packed state. The OH^−^ ions can then move faster between the anion membrane and the catalyst layer. This causes an increase in the availability of OH^−^ ions for a redox reaction to occur. At lower temperatures, the movement of hydroxide ions is slow due to a lower diffusion coefficient during electrolysis. This causes higher charge transfer resistance and thus reduces the availability of hydrogen ions to be released from the water-splitting process [37]. Increasing the temperature to more than 60 °C may adversely affect the membrane, due to the glass transition temperature of the AEM. To avoid membrane degradation, therefore, an optimum temperature of less than 60 °C should be maintained for the AEM electrolysis operation [38]. Figure 7 shows the energy consumption and efficiency for the different operating temperatures of the electrolysis cell. Based on Figure 7, when the temperature of the cell is increased, the energy consumed decreases, due to the increased energy efficiency of the cell.

**Figure 8:**
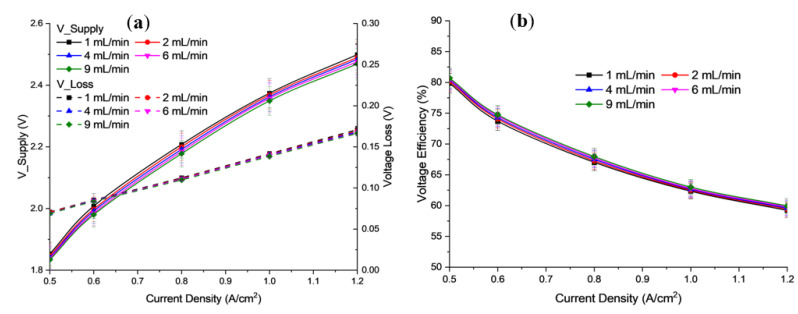
Profile of (**a**) Polarization curve, (**b**) Voltage efficiency for different electrolyte flow rate at 30 °C operational temperature and concentration of electrolyte 2.0 M KOH.

The results obtained are from the equations as described in the methodology, with current density ranging from 0.5 to 1.2 A/cm^2^ and operational temperatures from 30 to 60 °C. The temperature parameter has a significant effect on electrolysis performance based on energy efficiency. As shown in Figure 8, as temperature increases, the energy consumption is reduced, thus increasing energy efficiency. The temperature parameter at 60 °C shows the highest energy efficiency, while the lowest energy efficiency is at 30 °C. This is because, when the electrolysis process undergoes initial heating during start-up at a temperature of 30 °C, it requires more electrical energy. This electrical energy is then converted to chemical energy to be consumed in water electrolysis and is then wasted to heat energy based on the laws of thermodynamics. The optimum temperature, based on experimental analysis, is 60 °C, since it can produce high hydrogen production with the highest energy efficiency. This is because the AEM stack can utilize the high heat energy to increase the kinetic energy of water molecules, thus improving electrolysis efficiency and producing hydrogen at higher energy efficiency.

### 3.3. Effect of Flow Rate of Electrolyte

Figure 8a shows a diagram of the polarization curve, obtained from the results of the study, for the influence of flow rate. Figure 8b shows the voltage efficiency of potassium hydroxide (KOH) electrolyte for flow rates in the range of 1, 2, 4, 6, and 9 mL/min, at an operating temperature of 30 °C and 1 bar. Based on the results obtained, the flow rate of KOH electrolyte on the anode and cathode significantly affects the overall electrolysis efficiency. Both figures show that when current density increases, the cell voltage supplied decreases. This is also because when the flow rate of the electrolyte increases, the amount of cell resistance decreases. The obtained cell voltage of electrolysis at 1.2 A/cm^2^ was 2.469 V for 9 mL/min, while the cell voltage was 2.499 V for 1 mL/min. These results indicate that a higher flow rate of alkaline solution produces very good ionic conductivity of the membrane (in the MEA structure) [39].

**Figure 9:**
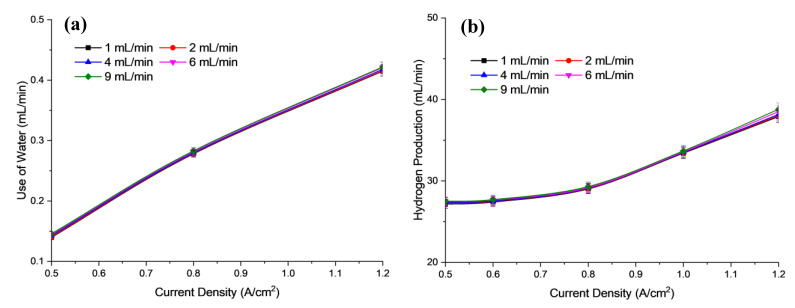
Profile of (**a**) use of water, (**b**) Hydrogen production based on electrolysis efficiency for different electrolyte flow rate at 30 °C operational temperature and concentration of electrolyte 2.0 M KOH.

Figure 9a shows the use of water while Figure 9b shows the hydrogen production at respective flow rates. The electrical conductivity of the electrolyte solution played an important role in the electrolysis system. This is evidence of the increase in electrolyte flow rate and current density contributing to the increase in the power supply. The increase in required input voltage has also led to increased electrical conductivity. This is because the rate of effective ionization has occurred. As a result, H_2_ gas production increases over time. Electrolysis depends not only on the number of ions present in the electrolyte solution but also on the mobility of the ions in the solution. In addition, increasing the flow rate of the electrolyte (KOH) will gradually increase the flow of ions in the solution, which subsequently decreases the mobility of the ions due to obstruction.

In AEM electrolysis, a KOH electrolyte solution, with water molecules carrying OH^−^ ions, is circulated to the anode side of the cell. The liquid electrolyte hydrates the membrane and the catalyst layer on the gas diffusion layer (GDL) without an additional supply of (excess) water. The flow of liquid electrolytes is very important as it is relates to the mass transfer of the redox reaction. The flow rate has a direct effect on the AEM electrolysis performance; hence, it is important to understand the effect of flow rate on the various resistances.

During the electrolysis operation, in the redox reaction, bubbles of oxygen form on the anode and bubbles of hydrogen on the cathode [40]. These products must be removed immediately from the surface of the GDL to prevent them from blocking the catalyst’s active sites. This can be achieved by increasing the flow rate of the electrolyte. Internal forces help to keep the two gases apart. On the other hand, a higher flow rate rapidly removes the OH^–^ ions, hence reducing the available reaction time for oxidation and reduction. This subsequently leads to lower availability of OH^–^ ions to the catalyst, and then a significant increase in use of water, thus increasing the hydrogen production at each flow rate.

**Figure 10:**
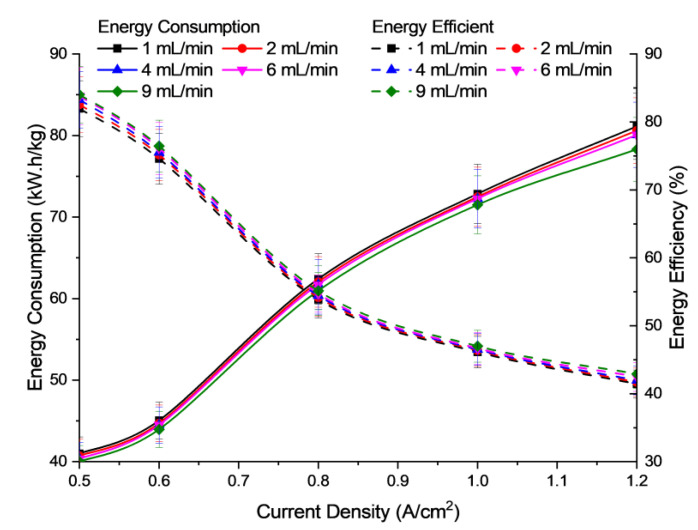
Profile of energy consumption and energy efficiency of electrolysis for different electrolyte flow rate at 30 °C operational temperature and concentration of electrolyte 2.0 M KOH.

Figure 10 shows the energy consumption and efficiency versus the electrolyte flow rate of the electrolysis cell. Based on Figure 10, the flow rate of electrolytes does not significantly affect the energy consumed or the performance of the cell unit. It is suggested that the electrolyte flow rate was supplied excessively, i.e., 1–9 mL/min for 5 cm^2^ effective area, and hence does not significantly affect the mass transfer limitation of the reactants, either in the form of convection or diffusion in the electrolyte cell. However, it is easily understood that the cell size and flow-field design could influence the electrolyte flow rate conditions and behaviour towards the cell performance.

As mentioned previously, the findings have shown that the use of electrolyte flow is a good method to improve cell performance and can be refilled in the electrolysis cell. Based on the used flow rate range for this study, it seems that it is no longer a limiting factor of reactant supply [41]. It means that the water supplied has exceeded the rate required by the AEM electrolysis cell for the electrolysis process to take place. Therefore, the use of an excessive flow rate only slightly affects the performance of the electrolysis [42].

### 3.4. Implications of Parameters on Electrolysis Performance

**Figure 11:**
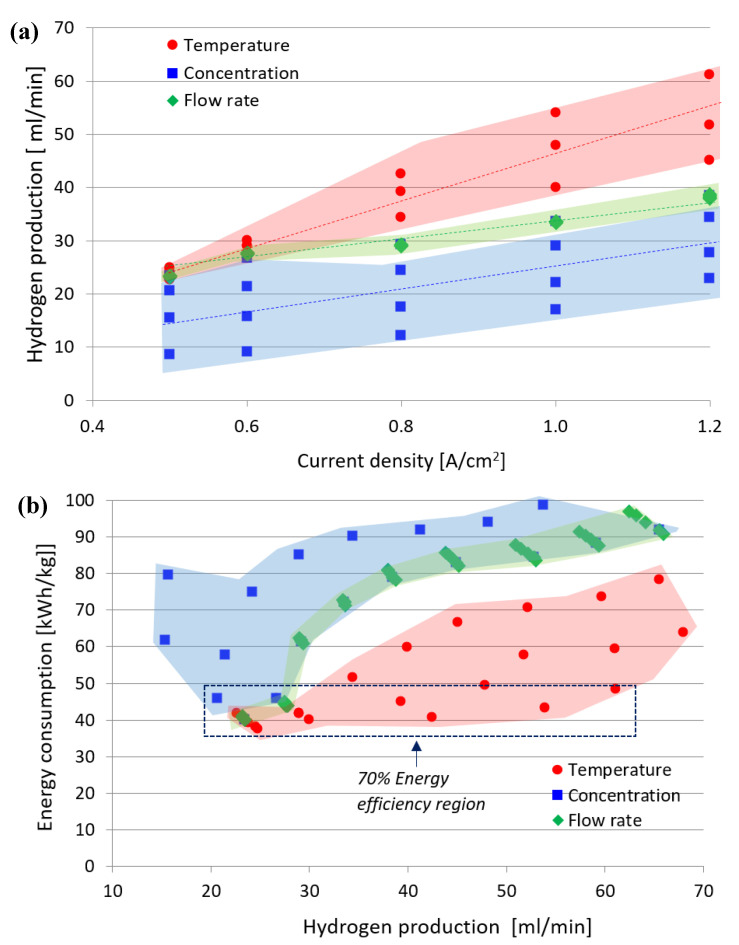
Summarized data for each operating condition: (**a**) Hydrogen flow rate, (**b**) Energy consumption for AEM electrolysis unit.

Figure 11a shows the energy consumption for the AEM electrolysis unit for each operating parameter based on the hydrogen flow rate. Figure 11b shows the hydrogen production rate based on the current supply. Based on the study conducted, the optimal conditions for the operation of the AEM electrolysis, offering high performance with low energy consumption and high hydrogen production, are as follows: 2 M KOH liquid electrolyte, a temperature of 60 °C, and a flow rate of 9 mL/min. Figure 11a shows the relationship between the hydrogen production flow rate to the applied current load at various operating parameters. In general, for all operating parameters, the flow rate of hydrogen increases with increasing load current. This is seen especially in temperature operations, where the region is large, and the slope is steeper and occupies the biggest area. The parameter involving the flow rate of electrolytes occupies the smallest region and thus shows little significance in the production of hydrogen.

Figure 11b shows the relationship between energy consumption (to produce 1 kg of H_2_) and the flow rate of hydrogen. In general, energy consumption decreases with increasing hydrogen production. To have a target and obtain energy efficiency above 70 %, energy consumption per 1 kg of H_2_ must be less than 47.5 kW·h/kg. Therefore, based on Figure 11b, the increase in operating temperature and some operating of either the flow rate or the concentration can increase the efficiency. However, this increase in hydrogen flow rate requires a high current load, as shown in Figure 11a. Based on Figure 11, to achieve energy efficiency above 70% for the low operating temperature, the current density is up to 0.6 A/cm^2^. While energy efficiency above 70 % for high operating temperature, i.e., 60 °C, could be achieved up to 1.2 A/cm^2^ with about 60 mL/min hydrogen production. Based on the relationship between cell voltage and current that has been discussed previously, an increase in current will increase the cell voltage and lower the cell voltage efficiency. With a decrease in voltage efficiency, the rate of cell degradation will also increase, further affecting the long-term hardening of the cell or system.

## 4. Conclusions

This study focused on the effects of operating conditions on single cell AEM electrolysis unit performance. The effects of the applied current (2.5 A to 6.0 A), potassium hydroxide (KOH) electrolyte concentration (0.5–2.0 M), electrolyte flow rate (1 mL/min to 9 mL/min), and operating temperature (30 °C to 60 °C), were determined. Based on the findings, the operating parameters greatly influence the performance of AEM electrolysis. For all operating parameters, the flow rate of hydrogen increases with increasing applied current. The operating temperature and electrolyte concentration significantly affect the electrolysis efficiency while electrolyte flow rate has a little significance. The optimal conditions for the operation of the AEM electrolysis, offering high performance with low energy consumption and high hydrogen production, are as follows: 2 M KOH liquid electrolyte, a temperature of 60 °C, and a flow rate of 9 mL/min. Hydrogen production of 61.13 mL/min is achieved with an energy consumption of 48.25 kW.h/kg and an energy efficiency of 69.64%. All these results indicate the importance of determining the effects of the operating parameters on the performance and durability of AEM type electrolysis to expand their application.

## Data Availability

All relevant data are contained in the present manuscript.
